# Domestic Correspondence

**Published:** 1889-09

**Authors:** 


### Domestic Correspondence.

To the Editor:

I send you a copy of the law which has just passed the legislature *repealing* our old law of 1885. As you see, it places a *premium* upon dental education procured from dental colleges, but, by asking in addition an *examination* of those holding diplomas, demands that the colleges also shall deal honorably by the profession and their students by giving no unmerited degrees. It also provides that in addition to the regular examination candidates shall be provided by the Board of Examiners with patients upon whom to perform actual dental operations: it also defines dentistry. We hope that this law may not only be of great service to the interests of the commonwealth of Minnesota, but that it will at the same time offer a stimulus to *worthy* dental colleges and a check to diploma mills.

J. H. Martindale, M.D.

To the Editor:

The sixth annual meeting of the Minnesota State Dental Association was held at the Spalding House, Duluth, July 10–12. There was a good attendance and an interesting programme. By way of entertainment the Duluth dentists gave the visitors a boat-ride on the lake, and Dr. Brown gave a reception at his residence.

The officers for the ensuing year are Dr. G. O. I. Brown, Duluth, President; Dr. L. C. Davenport, Moorhead, Vice-President; Dr. C. H. Robinson, Wabasha, Recording Secretary; Dr. M. G. Jenison, Minneapolis, Corresponding Secretary; Dr. H. M. Reid, Minneapolis, Treasurer.

The next annual meeting will be in Minneapolis, commencing the second Wednesday in July, 1890.

M. G. Jenison.

To the Editor:

A semi-annual union meeting of the Fifth, Sixth, Seventh, and Eighth District Dental Societies of the State of New York will be held in Elmira, N. Y., October 24 and 25, 1889. A large attendance is assured, and a meeting of unusual interest is expected. A more extended notice will appear later.

Myron D. Jewell.

